# Negative regulation of tumor-infiltrating NK cell in clear cell renal cell carcinoma patients through the exosomal pathway

**DOI:** 10.18632/oncotarget.16354

**Published:** 2017-03-18

**Authors:** Yang Xia, Qiongfang Zhang, Quan Zhen, Yan Zhao, Nanjing Liu, Ting Li, Yanni Hao, Yao Zhang, Chunli Luo, Xiaohou Wu

**Affiliations:** ^1^ Department of Urology, The First Affiliated Hospital of Chongqing Medical University, Chongqing 400016, China; ^2^ Key Laboratory of Molecular Biology for Infectious Diseases (Ministry of Education), Institute for Viral Hepatitis, Department of Infectious Diseases, The Second Affiliated Hospital, Chongqing Medical University, Chongqing 400016, China; ^3^ Key Laboratory of Diagnostics Medicine designated by the Ministry of Education, Chongqing Medical University, Chongqing 400016, China

**Keywords:** exosome, clear cell renal cell carcinoma, natural killer, transforming growth factor-β1, tumor immune evasion

## Abstract

Natural killer cells are the key components in tumor immunity and defects in function are necessary for tumor immune escape. Emerging studies on tumor cell-derived exosomes have shown the biological significance in tumor microenvironment, but the underlying role of exosomes in regulating natural killer cells functions in clear cell renal cell carcinoma patients remains unknown. Firstly, we precisely characterized the phenotype and function of natural killer cells in clear cell renal cell carcinoma patients vs healthy controls. With an inhibitory phenotype, tumor-infiltrating natural killer cells exhibited poor cytotoxic capacity and deficient potential to produce cytokines compared with natural killer cells from tumor margin tissue and non-tumor tissue. Next, we revealed that primary tumor cells trigged natural killer cell dysfunction in an exosome-dependent manner. Interestingly, exosomes from primary tumor cells were preferentially enriched with TGF-β1 which acted as important mediator of natural killer cell functional deficiency. *In vitro* culture of exosomes induced natural killer cell dysfunction mediated by activation of the TGF-β/SMAD signaling pathway, and abrogated by knockdown TGF-β. Our data indicate that exosomes from clear cell renal cell carcinoma induce natural killer cells dysfunction by regulating the TGF-β/SMAD pathway to evade innate immune surveillance.

## INTRODUCTION

Clear cell renal cell carcinoma (ccRCC) is the most common type of kidney cancer and follows an unpredictable disease course [1, 2]. It is very well established that ccRCC is an immunogenic tumor, due to the relatively high spontaneous regression rate and the high levels of tumor infiltrating immune cells. These tumor infiltrating immune cells, especially tumor-infiltrating natural killer (TINK) cells, are vital components in antitumor immunity. However, TINKs are reported to be influenced by the local cellular and soluble components of the tumor microenvironment and may be rendered dysfunctional, leading to tumor cells escaping from the host immune system [3, 4]. Therefore, it is of clinical interest to investigate how the tumor cells become resistant and escape from immune system control, and the cross-talk between tumor and immune cells.

NK cells play as first-line defenders in the host response to tumors [5]. Regulated by an integration of inhibitory and activating receptors, NK cells display at least two effector functions against the tumor cells: they can mediate direct cytotoxic activity through degranulation and they are also able to produce a variety of antitumor active and immunoregulatory cytokines such as IFN-γ, TNF-α [6]. NK cells are reported to participate in the destruction of various types of tumors [7–10]. However, their exact contribution to the control of human solid tumors, including ccRCC, remains disputed due to functional defects of TINK cells, resulting in tumor cells escaping from NK cell attack [11]. In this regard, the molecular events involved in the susceptibility of ccRCC cells to natural cytotoxic effectors should be taken into consideration.

Recently, a considerable interest in the cancer field has focused on the role of tumor-derived exosomes (Texs). Texs are tiny vesicles which characterized by a size of 30–150 nm in diameter, it became quite clear that these vesicles are important mediators of intercellular communication between tumor cells and immune cells [12, 13]. Texs generally modulates NK cells immune responses in a dual manner [14, 15]. The findings of Gastpar and colleagues showed that NK cells were stimulated by human pancreas and colon carcinoma cell-derived exosomes, in a Hsp70/Bag-4- dependent manner by engaging the activating NK cell receptor NKp30 and triggering the release of cytokines [16]. Lv LH [17] showed that incubation of NK cells with Hsp-bearing exosomes augmented cytolytic activity against K562 or HepG2 target cells through granzyme B release; up-regulation of activating receptors CD69, NKG2D, and NKp44; and down-regulation of inhibitory receptor CD94. However, several reports stated an immune suppressive effect of tumor cell-derived exosomes on NK cells. C. Liu [18] suggested that the treatment of mice with Texs decreased the percentage of NK cells in the spleen and lungs. Texs isolated from sera of patients with AML at the time of diagnosis had similar effects on the NK cells phenotype and function [19]. Because of limitation to obtain tumor tissue, most researches utilize cancer cell line to explore the function of exosome. However, there are differences in primary tumor cell cultures and cell lines. Primary culture of the whole cancer tissue derived from surgery or biopsy can indeed provide very important information as to our understanding of tumor properties.

In this study, we firstly isolated Texs from primary ccRCC cells to explore the crosstalk between Texs and NK cells in ccRCC tumor microenvironment. Here, we showed evidences of altered NK phenotype and function compared to controls in ccRCC patients. Then, we explored the immunoregulatory functions of exosomes on NK cells and found that ccRCC cell escape from NK cell–mediated immunity depend on exosomal mechanism. Finally, we provided evidences that ccRCC derived-exosomes enriched in TGF-β1 impaired TINK cell functions by activating the TGF-β/SMAD pathway. This finding may be of importance in the design of effective strategies that use exosomes for the treatment of clear cell renal cell carcinoma.

## RESULTS

### Patient characteristics

The demographic and clinical characteristics of the ccRCC patients and healthy control participants are given in the Table 1. The median age of the ccRCC patients was 60.9 years and the male to female ratio for ccRCC was 1:1.4.

**Table 1:** Clinical characteristics of patients and controls

	ccRCC patients	benign tumor patients	Healthy donors
N	36	16	36
Mean age (range)	60.9(35-88)	40.7(36-57)	34.5(12-43)
Gender			
Male	15(41.7%)	7(43.8%)	20(55.6%)
Female	21(58.3%)	9(56.2%)	16(44.4%)
TNM Stage			
I and II	28	_	_
III and IV	8	_	_
Tumor Size (cm)		_	_
<7	23	_	_
≥7	13		

### TINK cells in ccRCC display an abnormal phenotype

**Figure 1:**
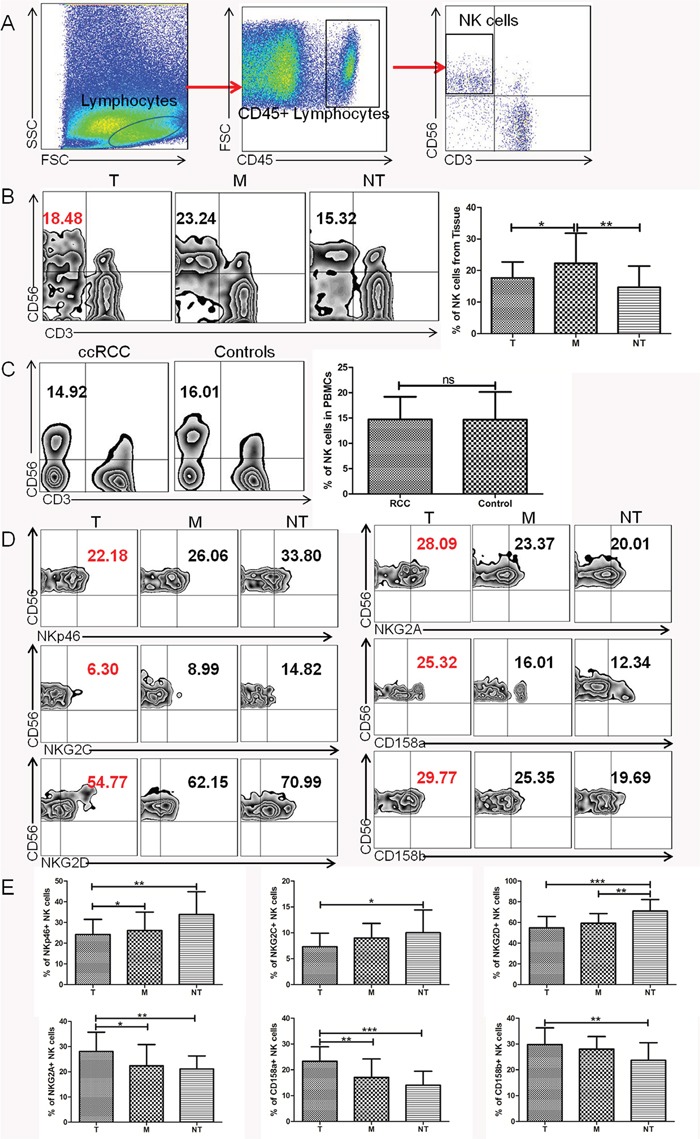
TINK cells in RCC display an abnormal phenotype **(A)** Lymphocyte gate of freshly purified mononuclear cells (MNCs) and NK cells. **(B)** Percentage of NK cells from tumor tissue (T, n = 36), tumor margin tissue (M, n = 36), and non-tumor tissue (NT, n = 16, mean±sd). **(C)** Percentage of NK cells in PBMCs from ccRCC patients (ccRCC, n = 36) and healthy individuals (HC, n = 36, mean±sd). **(D)** The percentage of NK-cell activation receptors NKp46, NKG2D, NKG2C and inhibitory receptors NKG2A, CD158a, CD158b expressed on NK cells from tumor tissue (T, n = 36), tumor margin tissue (M, n = 36), and non-tumor tissue (NT, n = 16, mean±sd). **(E)** Pooled percentage of NK receptors expressed on NK cells from tumor tissue, tumor margin tissue, and non-tumor tissue (T, n = 36; M, n = 36; NT, n = 16, mean±sd), *p < 0.05, **p < 0.01 and ***p < 0.001vs corresponding control.

Using flow cytometry, we analyzed NK cell frequency and phenotypic characteristics in ccRCC patients compared with controls. The gating strategy was presented in Figure 1A. The percentage of TINK cells was significantly lower in tumor tissue (T) compared to NK cells form tumor margin region (M) (P<0.05, Figure 1B) but markedly higher compared to NK cells from non-tumor tissue (NT) (P<0.01, Figure 1B). However, NK cells from PBMCs were comparable between ccRCC patients and healthy controls (Figure 1C). TINK cells had an abnormal phenotype, with lower frequencies of NK cells expressing the activating receptor NKp46, NKG2D, NKG2C (Figure 1D, 1E) and increased frequencies of NK cells expressing the inhibitory receptor NKG2A, CD158a and CD158b (Figure 1D, 1E) compared to NK cells from M region or NT tissue. There was no significant difference in the expression of NKp44, KIR2DL1/DS1 or KIR3DL1/DS1 KIRs among the three groups (data not shown). These data demonstrated that TINK cells from ccRCC patients displayed an inhibitory phenotype, suggesting an impaired function of these cells.

### TINK cells in ccRCC exhibit a functional deficiency and are associated with tumor progression

**Figure 2:**
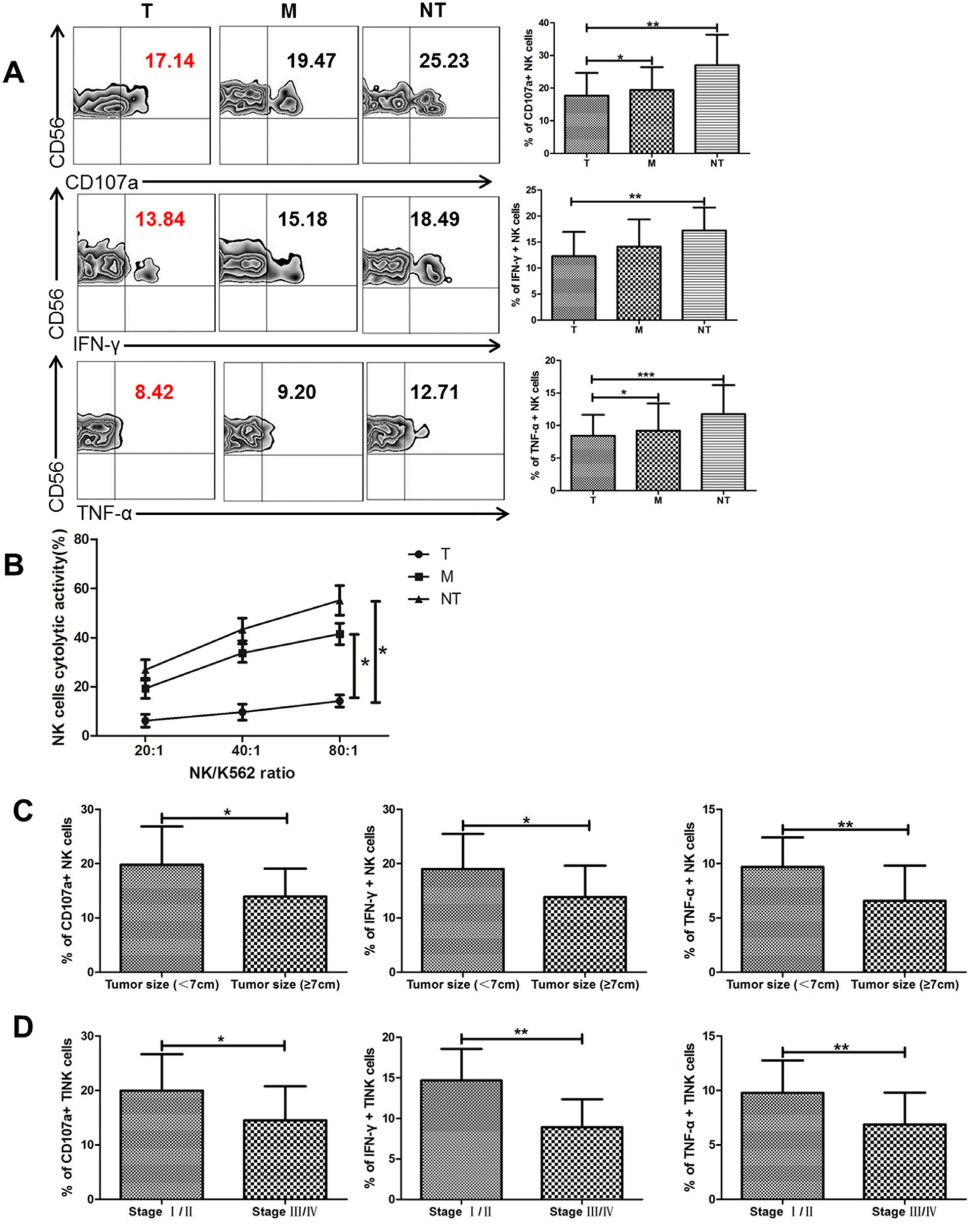
TINK cells in RCC exhibit a functional deficiency and are associated with tumor progression **(A)** The percentage of CD107a, IFN-γ and TNF-α expressed on NK cells from tumor tissue (T, n = 36), tumor margin tissue (M, n = 36), and non-tumor tissue (NT, n = 16, mean±sd). **(B)** The cytolytic activity of NK cells from different tissues cocultured with K562. **(C)** NK functional markers (CD107a, IFN-γ and TNF-α) expressed on TINK cells in patients with tumor diameters ≥7 cm (n = 13) VS patients with tumor diameters < 7 cm (n =23). **(D)** The percentage of CD107a, IFN-γ and TNF-α expressed on TINK cells decreases as tumors progress (n = 36). *p < 0.05, **p < 0.01 and ***p < 0.001vs corresponding control.

To further determine whether these phenotypic abnormalities were associated with impaired NK cell functions, NK-cell degranulation was evaluated by measuring the expression of CD107a, which was a functional marker for identifying NK cell-mediated lysis. We found that TINK cells from T regions were deficient in cytotoxic capacity with decreased expression of CD107a compared to NK cells from M regions and NT regions (Figure 2A). For capacity to produce cytokines, IFN-γ and TNF-α expression on NK cells were detected. There was a significant reduction in the levels of IFN-γ and TNF-α produced by TINK cells compared with other two groups (Figure 2A). We further validated whether degranulation and cytokine secretion correlated with target cell killing with modified lactate dehydrogenase (LDH) release assay using magnetically-purified NK cells (>90% purity) to kill K562 target cells. As shown in Figure 2C, TINK cells from ccRCC patients had a deficient ability to kill K562 targets compared with NK cells from M regions and NT regions (Figure 2B). We also evaluated the functional role of TINK cells on clinical outcomes. The clinical information of ccRCC patients in our study was presented in Table 1. As expected, significant defects of TINK cell functions were observed in patients with tumor diameters ≥ 7cm compared to patients with tumor diameters < 7cm. And the function of TINK cells decreased as tumors progressed, as illustrated in Figure 2D. Altogether, consistent with an inhibitory phenotype, TINK cells displayed impaired functions to degranulate and secrete cytokines and were associated with tumor progression.

### ccRCC mediated NK dysfunction and cytotoxicity impairment via exosomes

**Figure 3:**
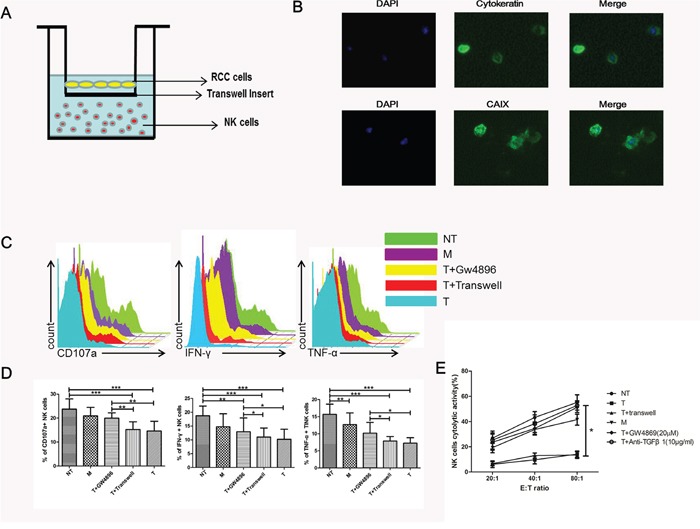
NK cell function was impaired via exosome **(A)** Transwell coculture model was used for the co-culture of primary NK cells and ccRCC cells. **(B)** Representative micrographs of immunofluorescence staining of cytokeratin and CAIX. DAPI counterstains nuclei in blue. 200× magnification. **(C, D)** NK cell functions (CD107a degranulation, IFN-γ and TNF-α production) in NT, M, T+GW4869, T+transwell and T groups. **(E)** Cytotoxicity of NK cells coculture with NT, M, T+GW4869, T+transwell, T and anti-TGFβ1 groups. All data shown were from three independent experiments. Error bars indicate ±SD, *p < 0.05, **p < 0.01 and ***p < 0.001vs corresponding control.

To clarify whether exosomes are involved in the process of deactivation of NK cells induced by ccRCC cells, we cultured purified NK cells and primary cells from NT, M and T tissues separately in transwells (Figure 3A). ccRCC primary cultures were established from surgical tissue specimens and assessed by immunofluorescence (Figure 3B). CAIX is a well-known marker of RCC and cytokeratin is the marker of tumor. By immunostaining with the cytokeratin and CAIX antibody, the ccRCC cells were confirmed. Primary tumor cells were grown at the top side of the cell culture insert (0.4 μm membrane pore size) of a Boyden (transwell) chamber and NK cells on the lower surface of the insert. With this experimental set-up, tumor cells could not pass through the chamber whereas exosomes were not affected, a direct transfer of exosomes from primary ccRCC cells to NK cells without direct cell-cell contact. As CD107a, IFN-γ and TNF-α expression are established markers of NK cell functional activity, we therefore assessed the expression of these markers of NK cells pretreated with different conditions. Figure 3C and 3D showed that ccRCC cells pretreated NK cells had significantly decreased levels of CD107a, IFN-γ and TNF-α as compared to NT tissue. Compared with the other group (ccRCC+transwell), cytolytic activity and functional activity of NK cells had no significant difference between the two groups (Figure 3C, 3D and 3E). GW4869, a well-known inhibitor of exosome secretion, was used to reduce the release of exosomes from ccRCC cells. When comparing ccRCC cells with ccRCC+GW4869, we found that that ccRCC cells could impair NK cells cytotoxicity and induce NK cells dysfunction, while GW4869 could attenuate this effect. It means ccRCC mediated NK dysfunction in a exosomal manner.

### Exosome purification and clinical significance

**Figure 4:**
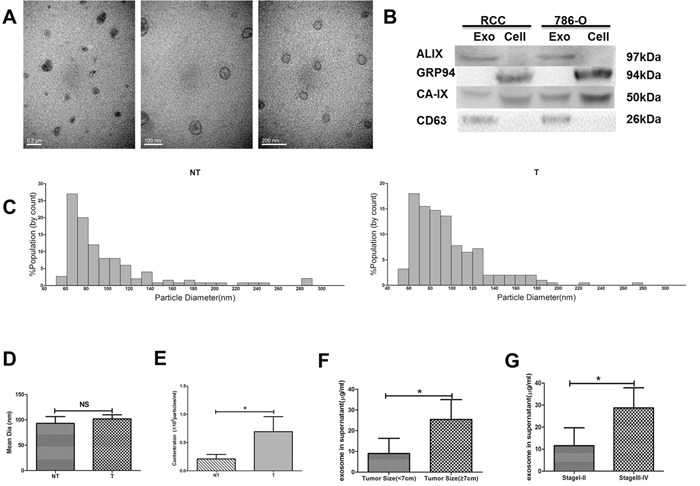
Exosome characterization **(A)** Transmission Electron Microscopy (TEM). **(B)** Western Blot analysis for exosomal marker in exosomes and cell lysate samples. **(C)** The quantification and size distribution analysis of exosomes. Size distribution of exosomes derived from tumor and non-tumor tissues were measured by qNano system. Bar Chart showing the mean diameter **(D)** and concentration for exosomes from NT and T **(E)**. Statistical analysis of the correlations between exosomal protein concentrations and clinical parameters, including tumor size **(F)** and TNM stage **(G)**. All data shown were from three independent experiments. Error bars indicate ±SD, *p < 0.05, **p < 0.01 and ***p < 0.001vs corresponding control.

Exosomes were isolated from primary cell culture supernatant of T, M and NT tissues. Transmission electron microscopy, western blot analysis and TRPS technology were used to characterize their integrity and morphology, purity and size distribution. TEM performed on exosomes derived from T region, M region and NT region revealed that all cells secreted vesicles displaying characteristic shape of exosomes (Figure 4A). There was no significant difference in the shape of exosomes derived from different regions. In addition, all kinds of markers were used to identify exosomes in primary ccRCC cells and human renal tumor cell line 786-O. ALIX and CD63 were often used as identification markers for exosomes. GRP94 was rarely expressed in exosomes but rich in all cells. CAIX was a well-known marker of RCC (Figure 4B). Exosomes were further characterized for size distribution analysis performed using TRPS technology. Exosomes from T region and NT region displayed sizes ranging between 50 and 150 nm (Figure 4C). It is interesting to note that T region derived Texs concentration is higher than NT region Texs, while the two kinds of exosomes showed the same size (Figure 4D and 4E). Furthermore, high levels of exosomal protein were positively correlated with advanced stage tumor and larger tumor size, as shown in Figure 4F and 4G.

### The immunesuppressive effects of ccRCC Texs

**Figure 5:**
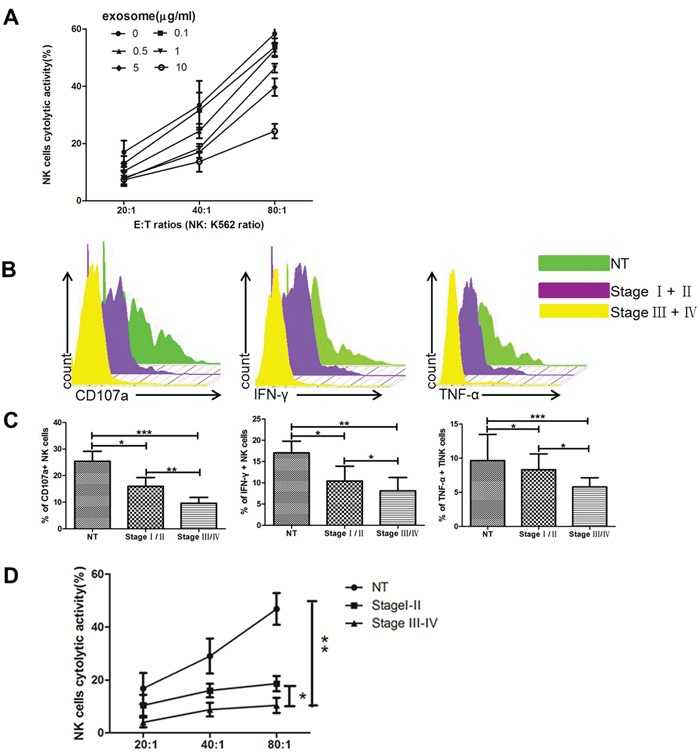
Texs treatment suppresses the cytolytic activity of NK cells *in vitro* **(A)** Cytotoxicity of NK cells coculture with Texs at different concentrations. **(B, C)** The level of CD107a, IFN-γ and TNF-α production of NK cells in coculture with Texs from different stages. **(D)** Cytotoxicity of NK cells coculture with Texs from different stages. All data shown were from three independent experiments. Error bars indicate ±SD, *p < 0.05, **p < 0.01 and ***p < 0.001vs corresponding control.

To examine the association between exosome expression in tumor region and clinicopathological parameters, we isolated primary NK cells pretreated with exosomes from ccRCC in different stages and NT region. Figure 5A showed that treatment of NK cells by Texs in different concentration decreased their cytotoxicity toward K562 target cells. The addition of tumor-derived exosomes strongly inhibited the NK cell- mediated killing of K562 target cells. Figure 5B and 5C showed stage III/IV Texs pretreated NK cells have significantly decreased levels of CD107a, IFN-γ and TNF-α as compared to stage I/II Texs and NT Texs. Figure 5D showed primary NK cells were incubated with different stages ccRCC Texs. Texs in stage III/IV showed even more defective NK cytotoxicity than that in stage I/II. Thus, Texs in stages III/IV are more suppressive than that in stage I/II. These results indicated that the decrease in the cytotoxicity and functions of NK cells was significantly higher when NK cells were pretreated with Texs from advanced stage tumor (stage III/IV) as compared to early stage (stage I/II).

### Exosomal TGF-β1 were associated with ccRCC progression

**Figure 6:**
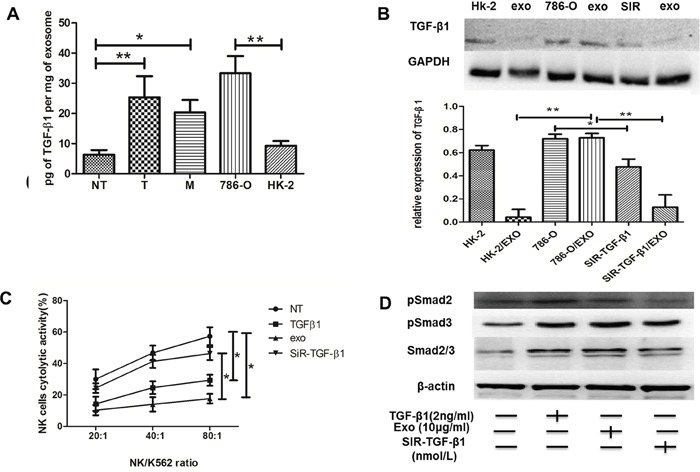
Texs interacted with NK cells through TGFβ1/Smad signaling **(A)** TGFβ1 were measured by ELISA in NT-exos, T-exos, M-exos, 786-O exos and HK-2 exos. High expressions of TGFβ1 protein were found in T-exos and 786-O. **(B)** Western blot of TGF-β1 protein. **(C)** Exos and NK cells were cocultured together to access the cytotoxicity of NK cells. Exos could impair the cytotoxicity of NK cell, and TGF-β1 knockdown could reverse it. **(D)** Knockdown of TGF-β1 could inhibit the smad signaling expression. All data shown were from three independent experiments. Error bars indicate ±SD, *p < 0.05, **p < 0.01 and ***p < 0.001vs corresponding control.

Previous studies have verified that cancer cells secreted TGF-β1 via exosomes [20]. However, the effect of TGF-β1 via exosomes in ccRCC cells was largely unknown. We then investigated whether exosomes affect the cytotoxicity of NK cells by a mechanism involving TGF-β1. From Figure 6A, we found the level of exosomal TGF-β1 derived from T region was obviously higher than that from NT region, while exosomes derived from renal cancer cell line 786-O were rich in TGF-β1 compared to proximal renal tubular cell HK-2. It meant that Texs increased TGF-β1 production. And the results were the same with the expression of TGF-β1 in western blot (Figure 6B). Interestingly, while 786-O cells were transfected with siR-TGF-β1, it showed lower expression of TGF-β1 in their exosomes in comparison with HK-2 exosomes. Consistently, exosomes derived from siR-TGF-β1 transfected 786-O cells reversed the decrease in the cytotoxicity of NK cells compared with that of exosomes secreted by 786-O cells (Figure 6C). These data demonstrated that negative regulation of NK cells by exosomes was mainly due to exosomal TGF-β1.

### Texs induced NK cell dysfunction via TGF-β1/smad signaling pathway

**Figure 7:**
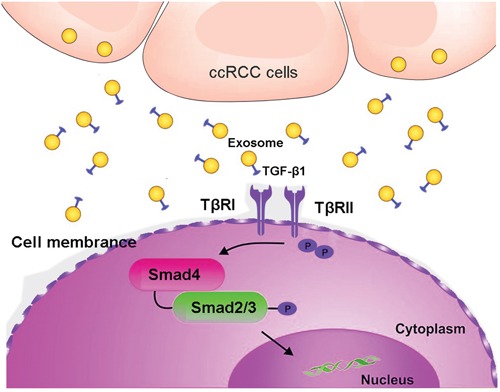
A schematic illustration showed that clear cell renal carcinoma cell-derived exosomes could induce NK cells dysfunction by activating Smad signaling through exosomal surface TGF-β1

We then evaluated the roles of exosomes in the activation of TGF-β signaling. Smad expression were promoted with TGF-β1 or Texs treatment (Figure 6D). NK cells co-cultured with Texs increased Smad2 and Smad3 phosphorylation, which are the most important signal transducers for transmission of TGF-β1 intracellular signaling. In addition, siR-TGF-β1 was used to partially suppressed the activation of TGF-β signaling (Figure 6D). As shown in Figure 6B, transfection with siR-TGF-β1 significantly down-regulated both intracellular and exosome levels of TGF-β1 in 786-O cells. Furthermore, the phosphorylation and total Smad2 and Smad3 protein expression were also decreased in treatment with exosome derived from siR-TGF-β1- transfected-786-O cells (Figure 6D). These results suggested that TGF-β1 in exosomes utilized the TGF-β1/SMAD pathway to inhibit NK cytotoxicity (Figure 7).

## DISCUSSION

Renal cell carcinoma (RCC) is an immunogenic tumor that characteristically harbors abundant infiltrating lymphocytes [3, 4]. NK cells are vital components in host defense against ccRCC [11]. TINKs are common features of ccRCC, but the immune responses of TINKs to tumor remain ineffective, and the exact mechanism underlying NK dysfunction in patients with ccRCC remains incompletely defined [21].

The phenotypic and functional profile of TINKs varies depending on the tumor subtype and the tumor microenvironment and may influence prognosis and disease outcome [22]. In colorectal cancer, tumor-infiltrating lymphocytes were associated with a favorable prognosis, however, this was not the case for ccRCC [23]. This fact led us to investigate the TINK cell frequency and expressions of NK cell receptors and NK function to comprehensively understand altered immune profiles of TINK cells in ccRCC. We found abundant NK cells in tumor region and tumor margin region compared to non-tumor region. Furthermore, abnormal phenotypic alteration of TINK cells evidenced by downregulation of activating receptors and upregulation of inhibitory receptors was also confirmed. We further detected NK functions in ccRCC patients vs controls. Consistent with an inhibitory phenotypic characteristic of TINK cells of ccRCC patients, profound defects of functions were found in NK cells from both primary tumor tissue and coculture models. Therefore, remolded by tumor microenvironment NK cells mediated antitumor immune response was impaired in ccRCC. This finding was consistent with several previous reports, which ultimately leaded to the establishment of an immunotolerant microenvironment [24, 25].

Recently, emerging evidences emphasize the role of exosomes secreted by various cancer cells in reprograming the microenvironment and supporting disease progression. Our results suggested that ccRCC cells utilized exosomes to suppress functions of their surrounding NK cells. Moreover, our results showed that Tex concentration was positively correlated with larger tumor size and advanced tumor stage in ccRCC patients. These findings not only indicate the inhibitory effect of exosomes in NK cells, but also suggest the clinical significance of exosomes in ccRCC patients.

In the following study, we focus on the exact role of ccRCC derived exosomes in the TINK-cell functions. Immunosuppressive factors, such as TGF-β1 and IL-10, were reported to alter NK cell functions in cancers [26]. Thus, we tested the levels of TGF-β1 and IL-10 in the supernatants collected from paired tumor and non-tumor primary renal cultures by ELISA. We found that increased levels of these two cytokines, most notably TGF-β1 in the supernatants collected from paired tumor and non-tumor primary renal cultures. These data confirmed that TGF-β1 might be the main immunosuppressive factor that caused the lack of activation of NK cells. In fact, the levels of TGF-β1 were also elevated in the exosomes of cancer patients and this was associated with systemic inhibition of immune function, including weakened NK cell responses [27, 28]. In this study, we measured the expression of TGF-β1 in exosomes, and found that ccRCC derived exosomes contained abundant TGF-β1 compared to those derived from normal cells. Then our studies validated that Texs promoted NK dysfunction through Smad signaling. In the study, TGF-β1 contained Tex contributed to activate TGF-β/Smad signaling pathway. To be added, we demonstrated that knockdown the TGF-β1 could improve the antitumor activity of NK cells *in vitro*. These data suggest that exosomes secreted by cancer cells promote NK cells dysfunction through the activation of TGF-β pathways.

In the past, many researches aiming to study TINKs were mainly focused on cancer cell line. We firstly utilized the primary ccRCC cell to explore the deficient function of TINK cells to escape from tumor surveillance. One limitation of the study should not be neglected that there was no data about survival rate of ccRCC patients. A survival rate during a follow-up period of 60 months will be presented to analyze the exact role of TINK cells and Texs enrich in TGF-β1 on clinical outcome in the future.

## CONCLUSIONS

This study demonstrates that clear cell renal carcinoma cell-derived exosomes could induce NK cells dysfunction by activating Smad signaling through exosomal surface TGF-β1 (Figure 7). These findings provide novel clarification of tumor immune evasion mechanisms which Texs may perform a suppressive role in ccRCCs by inducing NK cells dysfunction. These data will be helpful for potential targets for ccRCC immunotherapy.

## MATERIALS AND METHODS

### Patients

Peripheral blood and tumor tissues were collected from the Department of Urological Department of the First Affiliated Hospital of Chongqing Medical University. 36 ccRCC treatment naïve patients were enrolled in this study and all of the patients did not receive chemotherapy or radiotherapy. Detailed patient characteristics were shown in Table 1. Fresh tumor tissues, tumor margin tissues (at least 5 cm from the tumor margin) and peripheral blood samples of these 36 patients were used for further determinations. For controls, 16 kidney tissue samples were obtained from patients with benign kidney hemangioma and peripheral blood samples were obtained from 36 healthy individuals. All samples were collected after obtaining written informed consent from the donors and following approval by the Hospital Ethic Review Conmmitee of the First Affiliated Hospital of Chong Qing Medical University (register number 2016-68).

### Isolation of lymphocytes from samples

Freshly dissected tumor and non-tumor tissues were mechanically disrupted and then digested with 0.1% collagenase type IV (Sigma-Aldrich, St. Louis, MO) in serum-free RPMI 1640 for 1hour under agitation. After enzymatic digestion, single-cell suspensions were filtered and washed, and mononuclear cells (MNCs) were purified. Using Ficoll-Hypaque density gradient centrifugation, lymphocytes from tumor tissue, tumor margin tissue, non-tumor tissue and peripheral blood mononuclear cells (PBMCs) were isolated.

### Primary tumor cell culture

ccRCC tissue from nephrectomy specimens was minced, digested for 90 min at 37°C in 0.1% trypsin and 0.1% collagenase, washed, and cultured in RPMI-1640 medium. For each kidney, one central and at least one peripheral sample of tumor and one apparently uninvolved sample of tissue were used. By immunostaining with the cytokeratin and CAIX antibody, the ccRCC cells were confirmed.

### Flow cytometric analysis

Lymphocytes from blood/tissue samples were incubated for 30 minutes with the following mouse anti-human monoclonal antibodies: anti-CD45 (Biolegend, CA, USA); anti-CD3, anti-CD56, anti-perforine, anti-CD158a, anti-CD158b and anti-NKp44; anti-NKG2D, anti-NKp30, anti-CD226, and anti-NKp46 (BD Bioscience, San Jose, CA); anti-NKG2A and anti-NKG2C (R&D Systems, Inc., Minneapolis, MN); KIR2DL1/DS1 and KIR3DL1/DS1 (Beckman Coullter, Fullerton, CA). Mouse serum was used to block non-specific Fc receptor (FcR) binding, and isotype-matched IgGs were used as negative control antibodies. The samples were acquired using an fluorescence activating cell sorter (FACS) Calibur flow cytometer (BD Biosciences). Flow cytometry data were analyzed using FlowJo software (Tree Star, Inc., Ashland, OR).

### Degranulation and cytokine production of NK cells

For the detection of degranulation, freshly isolated lymphocytes were activated with 100 U/ml IL-2 (R&D Systems) overnight, and then cocultured with K562 cells at an effector to target ratio (E:T) of 10:1 for 2 hours. Anti-CD107a and monensin (10μg/mL; Sigma) were added directly into the medium and incubated for 4 hours. For the detection of cytokine production, isolated lymphocytes were directly stimulated with 50 ng/mL PMA (Sigma) plus 1 μg/mL ionomycin (Calbiochem, Darmstadt, Germany) for 2 hours and followed by the addition of monensin (10μg/mL; Sigma) (BD Sciences, SanJose, CA) for another 4 hours. Cells were then collected and stained with surface antibodies and stained intracellularly with anti-IFN-γ (eBioscience, San Diego, CA) and anti-TNF-α (Biolegend) in the dark. After incubation, the cells were washed, resuspended and finally detected by FACSCalibur (BD Bioscience).

### Cytotoxicity assay

According to the manufacturer's instructions, a commercially available lactate dehydrogenase (LDH) kit (Roche Diagnostics, Mannheim, Germany) was used with erythroleukaemic K562 cells (ECACC) as the target cell line and purified NK cells, which were activated with 100 U/ml IL-2 (R&D Systems) overnight, as effector cells. Target cells and effector cells were incubated for 2 h in 96 microwell plates (Flow, USA) with three effector: target (E: T) ratios of 80:1, 40:1 and 20:1 at 37°C with 5% CO2. Cell free supernatant was used to measure the level of released LDH as previously described [29].

### Co-culture experiments

NK cells were isolated from healthy blood using a Ficoll-Paque gradient and negative magnetic selection, as previously described [30]. NK cell purity was >90% as evaluated by FACS. Purified NK cells were cultured in RPMI 1640 supplemented with 10% FBS containing IL-15 (10 ng/ml; PeproTech) in 96-well cell culture cluster flat bottom plates either in the presence of primary cells from NT, M, T, T+transwell(96-well/1μm micropores, Millipore), T+GW4869 at a 20:1, 40:1, 80:1 ratio. Following the experiment, purified NK cells were coclutured with exosomes derived from NT, T in stage I/II and stage III/IV. After co-cultured for 48 hours, NK cells were harvested and analyzed. CD107a degranulation assay and intracellular cytokine staining for IFN-γ and TNF-α were usedto detect effector function.

### Preparation of exosomes and transmission electron microscopy (TEM)

All cells were grown at 37°C and 5% CO2 in non-FBS medium. Exosomes were isolated by differential centrifugation and were analyzed by TEM using negative staining method as previous described [31].

### Western blot

Protein extraction and Western blotting experiments were performed as described previously [32]. Primary antibodies used were as follow: anti-TGF-β1, CD63, ALIX, GRP94, CAIX (Abcam, Cambridge, MA), phospho-Smad 2, phospho-Smad 3, Smad 2/3, (Cell Signaling, Danvers, MA); and anti-β-actin, GAPDH (Sigma-Aldrich, St Louis, MO). HRP-conjugated horse anti-mouse and goat anti-rabbit IgG (Cell Signaling, Danvers, MA) were used as secondary antibodies.

### The quantification and size distribution analysis

The quantification and size distribution analysis of exosomes was performed using the Izon qNano system by TRPS technology (Izon Science Ltd, New Zealand) with the NP100 nanopore and 70 nm calibration beads as previously reported [33].

### ELISA

Concentrations of TGF-β1(R&D Systems, Inc., Minneapolis, MN) in the exosome from tumor region (T), tumor margin region (M), the non-tumor tissues (NT), cell line 786-O and HK-2 were determined using ELISA kits according to the manufacturer's instructions.

### Transfection with short-interfering RNA for TGF-β1

786-O cells were seeded at a concentration of 0.5×105 cells/ml (10-30% confluence) on the day before the transfection. Short-interfering RNA (siRNA) for TGF-β1 (siR-TGF-β1; Invitrogen, Carlsbad, CA) was used for the transfection of the cells, which was achieved by using cationic liposomes, Lipofectamine RNAiMAX (Invitrogen, Carlsbad, CA), according to the manufacturer's protocol. The nonspecific control siRNA (Shanghai Genechem Co., Ltd.) sequence was 5’-TTCTCCGAACGTGTCACGT-3’. The sequence of siR-TGF-β1 was 5’-caCACTGCAAGTGGACATCAA-3’. The effects manifested by the introduction of siR-TGF-β1 into the cells were assessed at selected time points after the transfection.

### Statistical analyses

Statistical analyses were conducted using Prism 5.0 (GraphPad Software, Inc.) All data are expressed as the mean ± SD. Student's t-test and one-way ANOVA were used to evaluate the significant associations among categorical variables. *p < 0.05, **p < 0.01 and ***p < 0.001 were considered statistically significant.

## Abbreviations

NK: natural killer
Texs: tumor cell-derived exosomes
ccRCC: clear cell renal cell carcinoma
TINK: tumor-infiltrating natural killer
TGF-β1: transforming growth factor-β1
PBMC: peripheral blood mononuclear cell
T: tumor
M: tumor margin region
NT: non-tumor tissues
CAIX: Carbonic anhydrase IX
TEM: transmission electron microscopy
TRPS: Tunable Resistive Pulse Sensing
MNCs: mononuclear cells.

## References

[1] Ljungberg B, Campbell SC, Choi HY, Jacqmin D, Lee JE, Weikert S, Kiemeney LA (2011). The epidemiology of renal cell carcinoma. European urology.

[2] Rini BI, Campbell SC, Escudier B (2009). Renal cell carcinoma. Lancet.

[3] Whiteside TL (2006). Immune suppression in cancer: effects on immune cells, mechanisms and future therapeutic intervention. Seminars in cancer biology.

[4] Webster WS, Lohse CM, Thompson RH, Dong H, Frigola X, Dicks DL, Sengupta S, Frank I, Leibovich BC, Blute ML, Cheville JC, Kwon ED (2006). Mononuclear cell infiltration in clear-cell renal cell carcinoma independently predicts patient survival. Cancer.

[5] Moretta L, Bottino C, Pende D, Mingari MC, Biassoni R, Moretta A (2002). Human natural killer cells: their origin, receptors and function. European journal of immunology.

[6] Guillerey C, Huntington ND, Smyth MJ (2016). Targeting natural killer cells in cancer immunotherapy. Nature immunology.

[7] Smyth MJ, Hayakawa Y, Takeda K, Yagita H (2002). New aspects of natural-killer-cell surveillance and therapy of cancer. Nature reviews Cancer.

[8] Wu J, Lanier LL (2003). Natural killer cells and cancer. Advances in cancer research.

[9] Miller JS, Soignier Y, Panoskaltsis-Mortari A, McNearney SA, Yun GH, Fautsch SK, McKenna D, TE Le C Defor, Burns LJ, Orchard PJ, Blazar BR, Wagner JE (2005). Successful adoptive transfer and *in vivo* expansion of human haploidentical NK cells in patients with cancer. Blood.

[10] Dutta A, Banerjee A, Saikia N, Phookan J, Baruah MN, Baruah S (2015). Negative regulation of natural killer cell in tumor tissue and peripheral blood of oral squamous cell carcinoma. Cytokine.

[11] Frankenberger B, Noessner E, Schendel DJ (2007). Immune suppression in renal cell carcinoma. Seminars in cancer biology.

[12] Bang C, Thum T (2012). Exosomes: new players in cell-cell communication. The international journal of biochemistry & cell biology.

[13] Hazan-Halevy I, Rosenblum D, Weinstein S, Bairey O, Raanani P, Peer D (2015). Cell-specific uptake of mantle cell lymphoma-derived exosomes by malignant and non-malignant B-lymphocytes. Cancer letters.

[14] Camussi G, Deregibus MC, Bruno S, Grange C, Fonsato V, Tetta C (2011). Exosome/microvesicle-mediated epigenetic reprogramming of cells. American journal of cancer research.

[15] Wang W, Lotze MT (2014). Good things come in small packages: exosomes, immunity and cancer. Cancer gene therapy.

[16] Pogge von Strandmann E, Simhadri VR, von Tresckow B, Sasse S, Reiners KS, Hansen HP, Rothe A, Boll B, Simhadri VL, Borchmann P, McKinnon PJ, Hallek M, Engert A (2007). Human leukocyte antigen-B-associated transcript 3 is released from tumor cells and engages the NKp30 receptor on natural killer cells. Immunity.

[17] Lv LH, Wan YL, Lin Y, Zhang W, Yang M, Li GL, Lin HM, Shang CZ, Chen YJ, Min J (2012). Anticancer drugs cause release of exosomes with heat shock proteins from human hepatocellular carcinoma cells that elicit effective natural killer cell antitumor responses *in vitro*. The Journal of biological chemistry.

[18] Liu C, Yu S, Zinn K, Wang J, Zhang L, Jia Y, Kappes JC, Barnes S, Kimberly RP, Grizzle WE, Zhang HG (2006). Murine mammary carcinoma exosomes promote tumor growth by suppression of NK cell function. Journal of immunology.

[19] Szczepanski MJ, Szajnik M, Welsh A, Whiteside TL, Boyiadzis M (2011). Blast-derived microvesicles in sera from patients with acute myeloid leukemia suppress natural killer cell function via membrane-associated transforming growth factor-beta1. Haematologica.

[20] Yamada N, Tsujimura N, Kumazaki M, Shinohara H, Taniguchi K, Nakagawa Y, Naoe T, Akao Y (2014). Colorectal cancer cell-derived microvesicles containing microRNA-1246 promote angiogenesis by activating Smad 1/5/8 signaling elicited by PML down-regulation in endothelial cells. Biochimica et biophysica acta.

[21] Shabtai M, Ye H, Frischer Z, Martin J, Waltzer WC, Malinowski K (2002). Increased expression of activation markers in renal cell carcinoma infiltrating lymphocytes. The Journal of urology.

[22] Eckl J, Buchner A, Prinz PU, Riesenberg R, Siegert SI, Kammerer R, Nelson PJ, Noessner E (2012). Transcript signature predicts tissue NK cell content and defines renal cell carcinoma subgroups independent of TNM staging. J Mol Med (Berl).

[23] Giraldo NA, Becht E, Remark R, Damotte D, Sautes-Fridman C, Fridman WH (2014). The immune contexture of primary and metastatic human tumours. Current opinion in immunology.

[24] Liu Y, Zhao L, Li D, Yin Y, Zhang CY, Li J, Zhang Y (2013). Microvesicle-delivery miR-150 promotes tumorigenesis by up-regulating VEGF, and the neutralization of miR-150 attenuate tumor development. Protein & cell.

[25] Muturi HT, Dreesen JD, Nilewski E, Jastrow H, Giebel B, Ergun S, Singer BB (2013). Tumor and endothelial cell-derived microvesicles carry distinct CEACAMs and influence T-cell behavior. PloS one.

[26] Vitale M, Cantoni C, Pietra G, Mingari MC, Moretta L (2014). Effect of tumor cells and tumor microenvironment on NK-cell function. European journal of immunology.

[27] Castriconi R, Cantoni C, Della Chiesa M, Vitale M, Marcenaro E, Conte R, Biassoni R, Bottino C, Moretta L, Moretta A (2003). Transforming growth factor beta 1 inhibits expression of NKp30 and NKG2D receptors: consequences for the NK-mediated killing of dendritic cells.

[28] Lee JC, Lee KM, Kim DW, Heo DS (2004). Elevated TGF-beta1 secretion and down-modulation of NKG2D underlies impaired NK cytotoxicity in cancer patients. Journal of immunology.

[29] Jurisic V, Spuzic I, Konjevic G (1999). A comparison of the NK cell cytotoxicity with effects of TNF-alpha against K-562 cells, determined by LDH release assay. Cancer letters.

[30] Pradier A, Passweg J, Villard J, Kindler V (2011). Human bone marrow stromal cells and skin fibroblasts inhibit natural killer cell proliferation and cytotoxic activity. Cell transplantation.

[31] Thery C, Amigorena S, Raposo G, Clayton A (2006). Isolation and characterization of exosomes from cell culture supernatants and biological fluids. Current protocols in cell biology.

[32] Zhang HG, Hyde K, Page GP, Brand JP, Zhou J, Yu S, Allison DB, Hsu HC, Mountz JD (2004). Novel tumor necrosis factor alpha-regulated genes in rheumatoid arthritis. Arthritis and rheumatism.

[33] de Vrij J, Maas SL, van Nispen M, Sena-Esteves M, Limpens RW, Koster AJ, Leenstra S, Lamfers ML, Broekman ML (2013). Quantification of nanosized extracellular membrane vesicles with scanning ion occlusion sensing. Nanomedicine (Lond).

